# Colorectal cancer at high risk of peritoneal metastases: long term outcomes of a pilot study on adjuvant laparoscopic HIPEC and future perspectives

**DOI:** 10.18632/oncotarget.17158

**Published:** 2017-04-17

**Authors:** Charlotte E.L. Klaver, Roos Stam, Didi A.M. Sloothaak, Johannes Crezee, Willem A. Bemelman, Cornelis J.A. Punt, Pieter J. Tanis

**Affiliations:** ^1^ Department of Surgery, Academic Medical Center, Meibergdreef 9, 1105 AZ Amsterdam, The Netherlands; ^2^ Department of Radiation Oncology, Academic Medical Center, Meibergdreef 9, 1105 AZ Amsterdam, The Netherlands; ^3^ Department of Medical Oncology, Academic Medical Center, Meibergdreef 9, 1105 AZ Amsterdam, The Netherlands

**Keywords:** colorectal cancer, peritoneal metastases, adjuvant HIPEC

## Abstract

**Objective:**

Early detection of peritoneal metastases (PM) of colorectal cancer (CRC) is difficult and treatment options at a clinically overt stage are limited. Potentially, adjuvant laparoscopic hyperthermic intraperitoneal chemotherapy (HIPEC) is of value. The aim of this study was to present long term oncological outcomes of a pilot study on adjuvant HIPEC to reduce development of PMCRC, with systematic review of literature.

**Methods:**

Long term oncological outcomes of ten patients who underwent laparoscopic HIPEC within eight weeks after resection of primary CRC in the pilot study were retrospectively collected. A systematic search of literature was performed on studies describing the use of HIPEC in patients with CRC at high risk of developing PM.

**Results:**

The median follow-up was 54 months (range 49-63). All patients were alive at the last follow-up moment and none of them had developed PM. Two patients had developed pulmonary metastases. Systematic review revealed five small cohort studies, including two matched comparisons. Peritoneal recurrences were found in 0% to 9% after adjuvant HIPEC, which was 28% and 43% in the two control groups, respectively. Disease free and overall survival were significantly higher in favour of HIPEC.

**Conclusion:**

Long term follow-up of ten patients included in a pilot study on adjuvant HIPEC revealed no peritoneal recurrences. This result is in line with other published pilot studies, a promising observation. However, the outcomes of the Dutch randomized COLOPEC trial and similar trials worldwide should be awaited for definitive conclusions on the effectiveness of adjuvant HIPEC.

## INTRODUCTION

The peritoneum is the third most common site of recurrence in colorectal cancer (CRC), and the incidence of peritoneal metastases (PMCRC) might even be higher than reported. This is because of the restricted sensitivity of imaging modalities for the small flat peritoneal lesions, which complicates the clinical diagnosis of PMCRC. The disease is often detected at a late symptomatic stage. Then, prognosis is poor and PMCRC seems to be relatively resistant to systemic therapy [1]–[5]. Only patients in good clinical condition with a limited extent of PMCRC are eligible for a curative intent treatment [6]. This consists of cytoreductive surgery with hyperthermic intraperitoneal chemotherapy (CRS/HIPEC), a procedure with a substantial risk of morbidity [7]–[9].

The difficulties with early detection of the disease, together with the restrictions of curative intent treatment options at a clinical overt stage, necessitate development of new therapeutic approaches for patients at high risk of PMCRC. Currently, prophylactic or adjuvant HIPEC and second look surgery, both aiming at treating PMCRC in an earlier (subclinical) phase, are under investigation [10]. Selection of high risk patients eligible for these approaches is based on risk factors identified in literature, including T4 stage [11]–[16], bowel perforation [16], [17], mucinous subtype [16], [18], and positive cytology of peritoneal lavage [16], [19]. Another relevant subgroup is the group of patients with already proven limited PMCRC that was resected together with the primary tumour or resected ovarian metastases. These high risk patients might be considered eligible for a second look strategy. Alternatively, prophylactic HIPEC at time of diagnosis might be considered [17].

In preparation of a randomized trial determining the effectiveness of adjuvant HIPEC, a single centre pilot study of ten high risk patients was performed in 2011 in order to determine the feasibility of adjuvant laparoscopic HIPEC in a short stay setting [20]. Feasibility criteria included: postoperative hospital stay of three days or shorter in at least six patients, a maximum of one conversion and a maximum of one re-admission within 30 days. These predefined feasibility criteria were fulfilled and adjuvant laparoscopic HIPEC was considered feasible. In the present analyses long term oncological outcomes of this pilot study are presented. In addition, an update of our systematic review of literature on adjuvant HIPEC [21] and an overview of other experimental strategies aiming at treating PMCRC in an earlier phase is given.

## RESULTS

### Update of the pilot study on adjuvant staged laparoscopic HIPEC

Baseline patient and disease characteristics are shown in Table 1. Nine patients were diagnosed with a T4 tumour, of which four were classified as T4b (tumour extension into adjacent organs/structures). At time of diagnosis nodal metastases were present in six patients, three patients already presented with omental metastases and one patient had simultaneous ovarian metastasis. The omental and ovarian metastases were resected simultaneous with the primary tumour. The type of primary tumour resection and additional resections for T4 stage or limited peritoneal metastases for each of the ten patients are displayed in Table 2. A primary anastomosis was made in all ten patients, with diverting ileostomy in one of these patients. The primary tumour resection was radical (R0) in seven out of ten patients. The median time interval between resection of the primary tumour and adjuvant laparoscopic HIPEC was six weeks.

**Table 1:** Patient and disease characteristics

	N = 10
Male: female	5:5
Age (median, years) [range]	59 [39–65]
ASA-score	
1	5
2	5
pT	
T3	1
T4a	5
T4b*	4
pN	
N0	4
N1/2	6
pM	
M0	6
M1**	4
Risk factor(s) for peritoneal metastases	
pT4 (only)	3
Omental metastasis	2
Omental metastasis and perforation	1
Ovarian metastasis	1
Obstruction/perforation	2
Positive lavage	1
Location of primary tumour	
Rectosigmoid	4
Transverse colon	2
Ascending colon	3
Caecum	1

ASA = American Society of Anesthesiologist; pTN = pathological TN. *In case of pathologic confirmation of tumour extension into adjacent organs/tissue, the tumour was classified as T4b. **M1: refers to resected solitary intraperitoneal metastases (ovarian / omental).

**Table 2:** Treatment characteristics

	Primary resection	Additional resections*	Radicality of resection	Interval between primary resection and HIPEC (weeks)	Interval between primary resection and adjuvant chemotherapy (weeks)	Adjuvant chemotherapy **
1	Right hemicolectomy	Partial small bowel, abdominal wall	R1	6	10	4 x CAPOX4x capecitabin monotherapy
2	Proctocolectomy	Omental metastasis	R0	4	10	12 x FOLFOX
3	Subtotal colectomy	Omentum	R0	3	7	5 x CAPOX, 3x capecitabin monotherapy
4	Right hemicoloectomy	Omental metastasis, psoas muscle	R1	7	10	12 x FOLFOX
5	Low anterior resection	Hysterectomy, bilateral sapingectomy, ileocecal resection, omentectomy	R0	3	10	8x CAPOX
6	Subtotal colectomy	No	R0	6	8	8 x CAPOX
7	Low anterior resection	Partial small bowel, abdominal wall	R0	6	8	8x CAPOX
8	Right hemicolectomy	Omental metastasis	R0	9	NA	Refused adjuvant systemic treatment
9	Right hemicolectomy	No	R0	6	12	6x FOLFOX
10	Low anterior resection	Ovarian metastasis	R1	4	8	8x CAPOX
				Median (range): 6(3-9)	Median (range): 10(7-12)	

CAPOX: combined schedule of capecitabin and oxaliplatin (standard 8 cycles); HIPEC = hyperthermic intraperitoneal chemotherapy; FOLFOX: combined schedule of 5-fluoruracil and oxaliplatin (standard 12 cycles); NA: not applicable; R0: microscopic complete tumour resection with all margins > 1mm; R1: microscopic margin involvement or margin ≤1mm.

*Additional resections, either of adjacent organs/structures because of suspected tumour ingrowth, or additional resections of metastases. ** Number of cycles and chemotherapeutic scheme.

All patients started with adjuvant systemic treatment, except for one patient who refused. Median interval between resection of the primary tumour and adjuvant systemic chemotherapy was ten weeks, with all patients starting within 12 weeks postoperatively. Six patients completed their adjuvant chemotherapy (four patients received CAPOX, two FOLFOX). In two patients, chemotherapy (CAPOX) was switched to capecitabin monotherapy after four and five cycles respectively, due to neurotoxicity and/or trombopenia. One patient completed only six cycles of FOLFOX, for unknown reasons. One patient experienced ascites after five cycles of systemic chemotherapy. Cytology did not reveal malignancy and ascites spontaneously disappeared. No further events were reported during adjuvant systemic chemotherapy.

**Figure 1:**
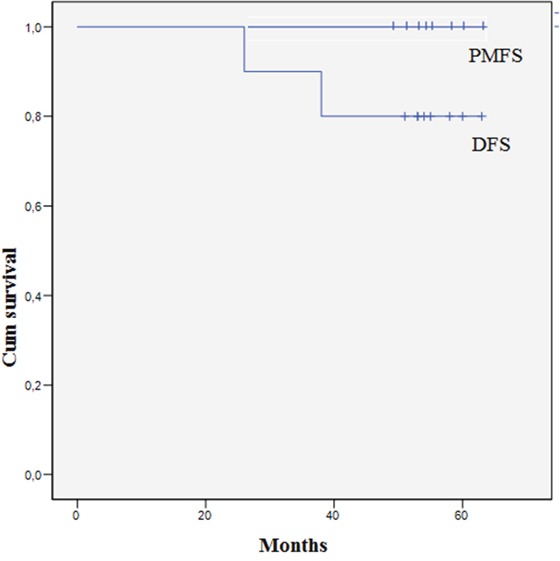
Disease free survival after adjuvant HIPEC PMFS: peritoneal metastases free survival. DFS: Disease free survival.

The median follow-up was 54 months (range 49-63). All patients were alive at the last follow-up moment and none of them has developed PMCRC (Figure 1). Two patients had disease recurrence, consisting of pulmonary metastases in both patients (Table 3), after 26 and 38 months respectively. Both patients underwent a microscopic irradical (R1) resection of the primary tumour (pT3N0 and pT4N1 respectively) and both had already intraperitoneal metastatic disease at time of resection of the primary tumour (an omental and an ovarian metastasis, respectively). One patient did not receive treatment for the pulmonary metastases but was carefully monitored and disease was stable at last follow-up. The other patient underwent a resection of the metastasis in the left upper lobe with curative intent and was disease free at last follow-up.

**Table 3:** Long term oncological outcome

	N= 10
Median follow-up (range)	54 (49-63)
Alive at end of follow-up	10/10
Local recurrence	0/10
Peritoneal metastases	0/10
Liver metastasis	0/10
Pulmonary metastasis	2/10
Other metastasis	0/10

### Update of the systematic review of literature

Repeating the literature search with restricted inclusion criteria revealed a total of five cohort studies on adjuvant HIPEC. One additional study of interest was published since our search in September 2013 [22], and the results of one study [23] were updated with long-term survival [24]. The inclusion criteria and treatment schedules are summarized in Table 4. Reported peritoneal recurrence rates until end of follow-up ranged from 0% to 9%. Two studies included a matched comparison with a control group of patients who underwent resection of the primary tumour followed by only adjuvant systemic therapy. In both studies, peritoneal recurrence rate, disease free survival, and overall survival were significantly different between the experimental and control group in favour of the intervention consisting of prophylactic target organ resection and simultaneous HIPEC at the time of primary tumour resection.

**Table 4:** Results of updated systematic review on adjuvant HIPEC in colorectal cancer patients at high risk of developing peritoneal metastases

author, year, design	inclusion criteria	n	HIPEC strategy + adjuvant systemic therapy	Overall/disease free survival	Peritoneal recurrence
Chouillard2009, single centre cohort[32]	colorectal cancer-T4,-pN2,-perforation,-positive peritoneal lavage,- peritumoral peritoneal nodules	16	Laparoscopic HIPEC, staged (median 5weeks, rang (0-8))MMC 80mg/m2, T=42-44°C t=35-45min	median FU 15.5 months2/16 died3/14 alive with metastasis	0% (median FU 15.5 months, range 8-29)
Lygidakis, 2010, single centre cohort [34]	rectal cancerN+, neurovascular involvement	87	Laparoscopic HIPEC, three procedures (22 days post-op, 25 days postop, 2 years postop)ip 5FU, Ox, LV, Ir; T=43C°; t=60min	1-year OS 100%	5% (2 of 40 patients who completed 2 years of follow-up and underwent the third laparoscopic HIPEC)
		(+4 cycles iv 5FU, Ox, LV, Ir)			
Tentes,2011, single centre cohort[33]	colorectal cancerT3/4	40	Open, simultaneous with tumour resection,MMC 15mg/m2 T= 42.5-43°C t=90minor Ox 130mg/m2 T= 42.5-43°C t=60min(+6 cycles iv 5FU/LV in stage III/IV)	actuarial 3-year OS100%	0% (median FU 17 months)
Sammartino, 2012, 2014matched comparison with control group[23], [24]	signet ring cell or mucinous colon cancerT3/4NxM0	25	Closed, simultaneous with tumour resection,prophylactic target organ resection ( appendectomy, omentectomy, resection of the round hepatic ligament and bilateral ovariectomy) Ox 460mg/m2 T=43°C t=30min, + iv 5FU + LV(iv 5FU/Ox (n=13))	median DFS: 36.8 monthsmedian OS: 59.5 months	4% (FU >48 months)
		50	(iv 5FU/Ox (n=23))	median DFS: 21.9 monthsmedian OS: 52 months	28% (FU >48 months)
				*p-value <0.05p-value < 0.04*	*p-value <0.03*
Baratti2016matched comparison with control group[22]	Colorectal cancer,- resected ovarian metastases,- minimal synchronous peritoneal disease (<1 cm in the omentum or close to the primary tumour),- T4a (n=8 vs. n=18)- T4b (n=9 vs. n=17)	22	Closed, simultaneous with tumour resection, prophylactic target organ resection (resection of the round hepatic ligament and lesser and greater omentectomy)Cisplatin 25mg/m^2^/L + MMC 3.3mg/m^2^/L T=42.5°C t=60min(iv 5FU/cap, FA, Ox (n=14), iv 5FU/cap, FA, Ox, Bev or Cet (n=4), 5FU/cap, FA, Ir, Bev or Cet (n=4))	5yOS: 81.3%5yPFS: 70.0%	5y cumulative incidence9.3% (median FU 65.2 months)
		44	(iv 5FU/cap, FA, Ox (n=29), iv 5FU/cap, FA, Ox, Bev or Cet (n=6), 5FU/cap, FA, Ir, Bev or Cet (n=6))	5yOS: 70.0%5yPFS: 18.3%	5y cumulative incidence42.5% median FU 34.5 months
				*p-value = 0.046p-value = 0.008*	*p-value <0.004*

Bev: bevacizumab; Cap: capecitabin; Cet: Cetuximab; DFS: disease free survival; FA: folinic acid; FU: follow-up; HIPEC: hyperthermic intraperitoneal chemotherapy; ip: intra peritoneal; Ir: irinotecan; iv: intravenous; LV: leucovorin; MMC: mitomycine-C; OS: overall survival; Ox: oxaliplatin; PFS: progression free survival; T: temperature of intraperitoneal infusion; t: duration of infusion;. 5y: 5 year; 5FU: fluorouracil.

## DISCUSSION

The present update of the small feasibility study of adjuvant laparoscopic HIPEC in preventing the development of PMCRC in high risk patients shows promising results. In none of the ten patients, peritoneal recurrence was detected after a median follow-up of 54 months, while in these high risk patients peritoneal recurrence rates between 14% and 58% are described in the literature [11], [19], [25]–[27]. Two patients developed distant metastases, of whom one underwent an intentionally curative resection. Moreover, all ten patients are still alive. Although the sample size is too small to draw conclusions, these results are in line with the encouraging results of five other studies investigating the role of adjuvant HIPEC in high risk CRC patients, as identified by systemic review of the literature (Table 4).

The interest in adjuvant HIPEC for high risk CRC is a revival of a treatment strategy that originates from the 1980’s. In the era of 5-FU as the only available cytotoxic agent for CRC, adjuvant intraperitoneal chemotherapy has been investigated, using administration of 5-FU through a peritoneal catheter in the immediate postoperative period or as prolonged treatment up to 12 months [28]–[31]. The update of our previously published systematic review reveals that the currently available evidence on adjuvant HIPEC is still restricted and consists of five small cohort studies. Follow up was relatively short in the three non-comparative series [32]–[34]. In the two other studies [7], [24] simultaneous HIPEC with so-called ‘target organ resection’ was compared to a matched control group and intermediate follow-up results have been published. A significantly lower incidence of peritoneal metastases in the experimental group was observed (4% vs. 28% and 9% vs. 43%, respectively), together with a significantly better survival (median OS: 60 vs. 52 months and 5yOS: 81% vs. 70%, respectively).

The initial results of our pilot study and the literature review supported the conduction of a randomized trial at that time, to determine the effectiveness of adjuvant HIPEC in preventing the development of PMCRC in high risk patients. Currently the COLOPEC multicentre randomized trial (NCT02231086) is recruiting. Included patients with T4 or perforated colon cancer are randomized between simultaneous or staged (5-8 weeks) open or laparoscopic HIPEC followed by adjuvant systemic chemotherapy, and a control arm of systemic chemotherapy alone [10]. Patient accrual is ahead of schedule and is expected to be finalized in the beginning of 2017. The primary outcome is peritoneal recurrence free survival after 18 months, which will be determined using laparoscopy in both study arms. Morbidity of adjuvant HIPEC is an important secondary outcome, considering the fact that the treatment is applied in a preventive setting. This means that the majority of patients undergo additional treatment without benefit, but with potential harm. Furthermore patients will be followed for a period of five years for (secondary) survival outcomes.

Presently, several randomized trials investigating proactive protocols to prevent PMCRC are performed. In the French ProphyloCHIP trial (NCT01226394), the effectiveness of second look laparotomy six months postoperatively with ‘in principle’ HIPEC is investigated. High risk is defined as perforated tumours, (resected) local peritoneal nodules and ovarian metastases, although the latter two should actually be considered as already proven PMCRC. Patient accrual of this trial has been completed. A similar phase III trial started in October 2014 at the Zhejiang University, China, with second look laparotomy and HIPEC six months postoperatively, including also pT4 cancers in addition to the other three inclusion criteria of the French trial (NCT02179489). In Italy, a currently recruiting randomized trial investigates the role of second look surgery six months postoperatively in mucinous CRC (NCT01628211). Based on the results of the matched comparative study, Sammartino initiated the PROMENADE trial (NCT02974556), which will start in 2017. This trial randomizes between simultaneous adjuvant HIPEC (oxaliplatin) and target organ resection (omentectomy, bilateral adnexectomy in post-menopausal patients, appendectomy and hepatic round ligament resection), and a control group with only adjuvant systemic chemotherapy. At the 10^th^ international Congress on Peritoneal Surface Malignancies (November 2016, Washington), an almost similar Spanish multicentre study was presented, which already started accrual (NCT02614534). Inclusion in this trial is based on imaging with selection of only ‘clear’ cT4 stage colon cancer. In contrast to the PROMENADE trial, the Spanish trial also performs target organ resection, but without HIPEC, in the control arm.

The different approaches in the trials that are currently in conduct, reveal one of the key questions in adjuvant intraperitoneal treatment for patients at high risk of developing PMCRC; namely the timing of the intervention. Proposed strategies vary from HIPEC simultaneous with the resection of the primary tumour, to staged HIPEC (several weeks postoperatively), to second look surgery six to twelve months postoperatively with prophylactic HIPEC depending on intraoperative findings.

Patient inclusion essentially differs based on the chosen strategy of adjuvant HIPEC. Selection should be based on clinical criteria if HIPEC is performed simultaneous with primary resection. This includes pre-operative imaging, histological biopsies and intra-operative findings. However, based on our experience, it is difficult to adequately select patients based on clinical staging. A clear cT4 stage based on imaging or intraoperative findings quite frequently turns out to have a pathological T3 stage. Also, a patient with a T4 tumour may postoperatively be classified as having stage II disease with microsatellite instability, which is regarded as low risk disease. According to the current Dutch guidelines, this is even not an indication for adjuvant systemic chemotherapy. Contrarily, a small area of peritoneal penetration (pT4a) is only diagnosed after scrutinizing the resection specimen by the pathologist. Furthermore, centres that do not perform HIPEC procedures have to refer their patients after the primary resection, leaving a staged HIPEC procedure as the only option.

The obvious advantage of a simultaneous approach is that no secondary surgical procedure is required. Also, it is hypothesised that free intraperitoneal tumour cells become encapsulated with fibrin, which makes these cells less accessible for chemotherapy at a later stage. A hypothetical disadvantage of the staged procedure is the delay in adjuvant chemotherapy, which potentially increases the risk of distant metastases. Following the Dutch guidelines, adjuvant chemotherapy should be administered not later than 12 weeks after the primary resection, and most oncologists strongly prefer to start within eight weeks. However, the exact effect of delaying adjuvant systemic chemotherapy remains unclear, because most studies on this topic are subject to a selection bias. It is difficult to conclude whether worse oncological outcomes should be ascribed to the delay in chemotherapy or to the underlying cause of the delay (postoperative complications, worse patient condition and/or comorbidities). The COLOPEC trial might provide the first comparative results that address the question of the effect of delayed adjuvant chemotherapy.

A second disadvantage of performing HIPEC several weeks after the resection of the primary tumour is the potential presence of intraabdominal adhesions that could cause difficulties in gaining access to the abdominal cavity and/or require adhesiolysis. However, both the present study and the published French study on staged adjuvant laparoscopic HIPEC revealed no conversions, even in patients who underwent open resection of the primary tumour [20], [32]. Adhesion scores are carefully registered during the staged HIPEC and the routine laparoscopy 18 months postoperatively to address this question in the COLOPEC trial.

Another option is second look surgery after six to twelve months, thereby not interfering with the standard resection and adjuvant systemic chemotherapy. However, disease progression might have occurred after six months, leading to the necessity of cytoreductive surgery in case of PMCRC. Results of the afore mentioned trials should be awaited to identify the most adequate approach, taking into account both the effectiveness and the extra morbidity of these preventive strategies.

In conclusion, pilot studies on adjuvant HIPEC in patients at high risk of developing PMCRC show promising results. These results have to be confirmed by currently recruiting randomized studies. Optimal patient selection and timing of prophylactic HIPEC are some of the issues that have to be resolved in the future.

## MATERIALS AND METHODS

### Update of the pilot study on adjuvant staged laparoscopic HIPEC

Between January 2011 and July 2012, ten patients were included in a single centre pilot study, with a diagnosis of adenocarcinoma of the colon and proximal rectum and at least one of the following risk factors for PMCRC: pT4, (resected) local peritoneal nodules in the close proximity of the primary tumour, primary tumour presenting with obstruction and/or perforation, positive cytology in peritoneal lavage, ovarian metastasis or omental metastasis [20]. More detailed in- and exclusion criteria have been published previously [20].

Included patients were planned to undergo a laparoscopic HIPEC procedure within 4 to 8 weeks after resection of the primary tumour. Perfusion with mitomycin-C (35mg/m^2^) was performed for 90 minutes at a flow rate of 1-2L/min with an inflow temperature of 42-43°C. A detailed description of the laparoscopic HIPEC procedure can be found in the original report of the pilot study [20].

In the original pilot study, median length of follow-up of patients was 13 months (range 10-26). For the present analysis, data on long term oncological outcomes were collected retrospectively. All patients intentionally received follow-up according to the Dutch guidelines, with outpatient clinic visits every 6 months in the first two to three years and yearly thereafter, CEA measurements every 3 to 6 months in the first 2 years and every 6 to 12 months thereafter, and liver ultrasound or CT abdomen every 6 months in the first two years and yearly until 5 years after primary diagnosis.

For the original pilot study, approval was obtained from the Institutional Review Board at the Academic Medical Center, Amsterdam, the Netherlands. Patients consented to collection of anonymised long term data. Therefore, no separate ethical approval for the present study was obtained.

### Update of the systematic review of literature

An update of our previously published systematic review of literature on intraperitoneal chemotherapy as adjuvant treatment to prevent PMCRC was performed [21]. The systematic search of published literature in Pubmed, Embase and the Cochrane database was repeated in November 2016 using the original search terms (Supplementary Figure 1).

Case series (n≤5) were excluded, as well as non-English language. For the purpose of the present update, eligibility of identified studies was restricted compared to the original review. Only studies describing the use of HIPEC in patients with CRC at high risk of developing PM were considered eligible for full text assessment. Furthermore, studies were included if primary data was provided on survival and/or peritoneal recurrence.

## SUPPLEMENTARY MATERIALS FIGURE



## Abbreviations

CRC: colorectal cancer
CRS: cytoreductive surgery
DFS: disease free survival
FU: follow-up
HIPEC: hyperthermic intraperitoneal chemotherapy
PMCRC: peritoneal metastases of colorectal origin
pTN: pathological TN
OS: overall survival
PFS: progression free survival
R0: microscopic complete tumour resection with all margins > 1mm
R1: microscopic margin involvement or margin ≤1mm
5y: 5 year.
