# Supplementary material for: Colorectal cancer at high risk of peritoneal metastases: long term outcomes of a pilot study on adjuvant laparoscopic HIPEC and future perspectives
Source: Oncotarget. 2017 Apr 17;8(31):51200–9. doi: 10.18632/oncotarget.17158 (PMC5584242; doi:10.18632/oncotarget.17158)
Supplement: Supplementary file 1 [file oncotarget-08-51200-s001.pdf]

Colorectal cancer at high risk of peritoneal metastases; long term outcomes of a pilot study on adjuvant laparoscopic HIPEC and future perspectives

SUPPLEMENTARY MATERIALS

SUPPLEMENTARY FIGURE

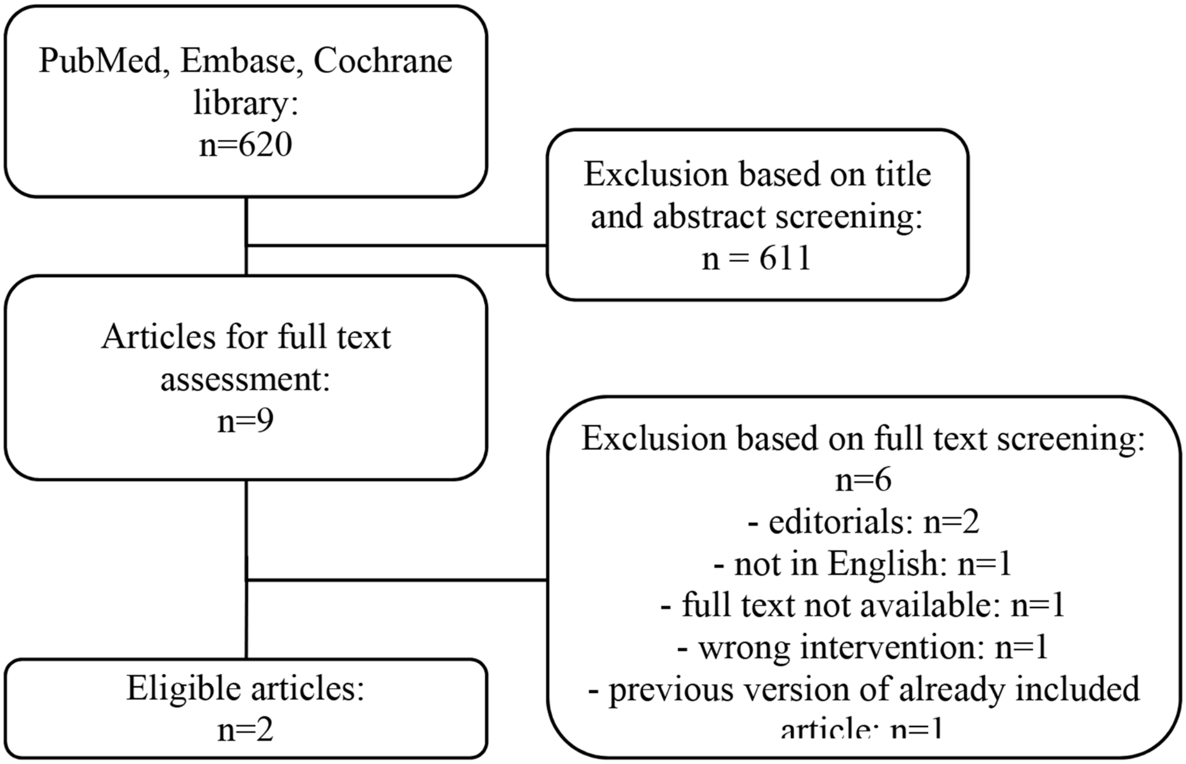

Supplementary Figure 1: Flow chart of search results from August 2013-November 2016.
